# Bariatric Surgery–How Much Malabsorption Do We Need?—A Review of Various Limb Lengths in Different Gastric Bypass Procedures

**DOI:** 10.3390/jcm10040674

**Published:** 2021-02-10

**Authors:** Daniel Moritz Felsenreich, Felix Benedikt Langer, Jakob Eichelter, Julia Jedamzik, Lisa Gensthaler, Larissa Nixdorf, Mahir Gachabayov, Aram Rojas, Natalie Vock, Marie Louise Zach, Gerhard Prager

**Affiliations:** Division of General Surgery, Department of Surgery, Vienna Medical University, 1090 Vienna, Austria; moritz.felsenreich@meduniwien.ac.at (D.M.F.); felix.langer@meduniwien.ac.at (F.B.L.); jakob.eichelter@meduniwien.ac.at (J.E.); julia.jedamzik@meduniwien.ac.at (J.J.); lisa.gensthaler@meduniwien.ac.at (L.G.); larissa.nixdorf@meduniwien.ac.at (L.N.); gachabayovmahir@gmail.com (M.G.); aram030884@gmail.com (A.R.); natalie.vock@hotmail.com (N.V.); n1507743@students.meduniwien.ac.at (M.L.Z.)

**Keywords:** malabsorption, Roux-en-Y gastric bypass, one-anastomosis gastric bypass, SADI-S, biliopancreatic diversion, weight regain

## Abstract

The number of obese individuals worldwide continues to increase every year, thus, the number of bariatric/metabolic operations performed is on a constant rise as well. Beside exclusively restrictive procedures, most of the bariatric operations have a more or less malabsorptive component. Several different bypass procedures exist alongside each other today and each type of bypass is performed using a distinct technique. Furthermore, the length of the bypassed intestine may differ as well. One might add that the operations are performed differently in different parts of the world and have been changing and evolving over time. This review evaluates the most frequently performed bariatric bypass procedures (and their variations) worldwide: Roux-en-Y Gastric Bypass, One-Anastomosis Gastric Bypass, Single-Anastomosis Duodeno-Ileal Bypass + Sleeve Gastrectomy, Biliopancreatic Diversion + Duodenal Switch and operations due to weight regain. The evaluation of the procedures and different limb lengths focusses on weight loss, remission of comorbidities and the risk of malnutrition and deficiencies. This narrative review does not aim at synthesizing quantitative data. Rather, it provides a summary of carefully selected, high-quality studies to serve as examples and to draw tentative conclusions on the effects of the bypass procedures mentioned above. In conclusion, it is important to carefully choose the procedure and small bowel length excluded from the food passage suited best to each individual patient. A balance has to be achieved between sufficient weight loss and remission of comorbidities, as well as a low risk of deficiencies and malnutrition. In any case, at least 300 cm of small bowel should always remain in the food stream to prevent the development of deficiencies and malnutrition.

## 1. Introduction

Obesity is an increasingly important disease considering the ever-growing numbers of patients worldwide. The World Health Organisation (WHO) has defined obesity as a body mass index (BMI) of >30 kg/m^2^ [1]. It massively impairs patients’ quality of life as well as their life expectancy, and has turned out to be a chronic disease that is difficult to cure [2]. The goal of a successful treatment is to achieve long-term weight loss and a remission or improvement of related comorbidities. Due to the chronic character of obesity, almost all conservative treatments for weight-loss are impermanent, and patients usually regain their body weight within a short period of time after an initial weight loss. Conservative treatments for obesity are dietary changes, behaviour therapy, physical activity or medication (e.g., Liraglutide, a glucagon-like peptide-1 receptor agonist). Furthermore, endoscopic approaches, such as gastric balloons or endoscopic suturing techniques, are options as well. Nevertheless, currently, the best option for obese patients to achieve long-term weight loss is bariatric surgery [3].

Procedures combining restriction with malabsorption tend to be superior in the long-term follow-up when compared to restrictive procedures [4]. So far, studies with up to 20 years follow-up after various bariatric procedures have reported a relatively stable weight after surgery [5]. Nevertheless, up to 40% of the patients experience weight regain (WR) after bariatric surgery with the need of an additional intervention. The manifestation of WR depends on its definition, the chosen bariatric procedure and the length of the follow-up [6].

Therefore, it is important to find the right procedure for the individual patient to obtain long-term weight loss with only a small risk of WR [7]. Further considerations when selecting a bariatric procedure, or when choosing the individual limb lengths in bypass procedures, should focus on the patient’s individual comorbidities. The choice of procedure always entails a balance between weight loss and remission of comorbidities on the one hand, and a risk of deficiencies (that should be minimal) on the other.

When talking about limb lengths in this context, it is important to consider that the total length of the human small bowel varies immensely between individuals. An analysis of ten studies measured the small bowel of 443 patients and found a mean length of 690 cm ± 93.7, with an enormous range of 350 cm to 1049 cm [8]. Not only sex and height of the patient, but also the technique of measurement, may influence the result [8,9].

## 2. Inclusion of Malabsorptive Bariatric Procedures

This article aims at reviewing the most frequently performed bariatric procedures worldwide that have been recognized by the International Federation for the Surgery of Obesity (IFSO) in terms of malabsorption. These procedures are Roux-en-Y-Gastric Bypass (RYGB), One Anastomosis Gastric Bypass (OAGB) and Single Anastomosis Duodeno-Ileal Bypass + Sleeve Gastrectomy (SADI-S). Biliopancreatic Diversion with Duodenal Switch (DPD + DS) was included as well, even though it only makes up to 0.5% of all bariatric procedures worldwide. However, it is the most common example of a procedure with a very strong malabsorptive component. 

The present article also discusses the issue of revisional procedures that add malabsorption to reinitiate weight loss in patients with weight regain after primary bariatric procedures. Adding malabsorption to a primary procedure bears a potential risk of deficiencies alongside the advantage of further weight loss [10]. Most studies on this topic available today have looked at revisional procedures after RYGB.

The most common bariatric procedure, Sleeve Gastrectomy (SG), as well as the formerly very common Gastric Banding (GB), will not be discussed as both procedures are based on the principle of restriction instead of malabsorption. Endoscopic malabsorptive procedures, such as the Endobarrier^®^, GI-window^®^, among others, were not included either due to the small number of patients or the short lengths of follow-up in most studies available today [11].

This review article focusses on high-quality comparative studies of the discussed procedures. However, it does not claim to cover all studies available in the literature. In order to work out the impact of different limb lengths in a bypass procedure, even studies on extreme variants (i.e., DPD, DPD + DS) were included in this review article. As the heterogeneity of the included studies varied in terms of outcomes, type of study, length of follow-up etc., a common endpoint could not be defined for this article.

## 3. Roux-en-Y-Gastric Bypass

**Figure 1:**
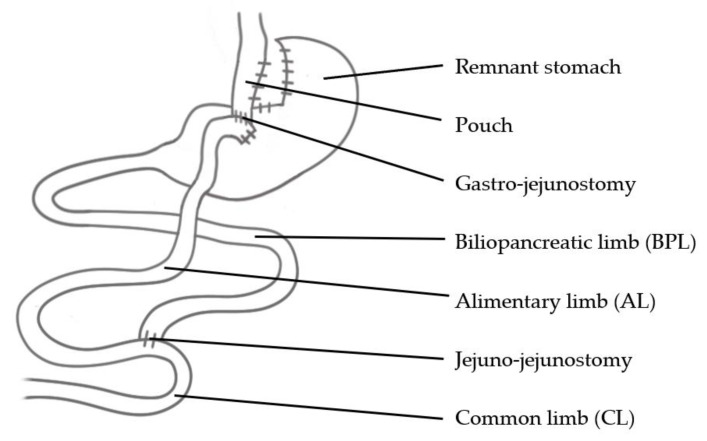
Roux-en-Y-Gastric Bypass (RYGB)/Diverted One-Anastomosis Gastric bypass (D-OAGB).

RYGB, the most common bariatric procedure relying on malabsorption, is a gastric bypass featuring a gastric pouch, a gastro-jejunostomy and a jejuno-jejunostomy (Figure 1). Various techniques have been used to perform a RYGB; differences mainly lie in the lengths of the alimentary limb (AL), the biliopancreatic limb (BPL) and the common limb (CL). These variations are, e.g., Long-Limb Gastric Bypass, Short-Limb Gastric Bypass, classic (standard) RYGB, Distal Gastric Bypass, Very-Long-Limb Gastric Bypass and Diverted One-Anastomosis Gastric Bypass (D-OAGB). Even within these categories, limb lengths and pouch sizes are not strictly defined and vary from one study to another [12]. These differences may certainly complicate comparisons in terms of malabsorption. However, this chapter aims at discussing research on the lengths of AL, BPL and CL to highlight the proportions ensuring sufficient weight loss paired with a low risk of malnutrition. 

Classic (or standard) RYGB, which is the bypass procedure most studied in the literature, is performed creating an AL of 150 cm, a BPL of 40–70 cm and a CL varying in length. Traditionally, the pouch is small and short. The malabsorptive component of this RYGB is minor (similar to Short-Limb Gastric Bypass), since only 40–70 cm BPL is excluded completely from the food stream, whereas in the 150 cm of AL only the uptake of lipides and triglycerides is excluded [13]. RYGB is usually indicated for patients suffering from gastro-esophageal reflux disease [14].

Short-Limb Gastric Bypass was described to only include 10 cm of BPL and 40 cm AL, and Long-Limb Gastric Bypass as 100 cm BPL and 100 cm AL in a publication by Christou N. et al. [15]. The outcomes of these procedures were compared in 228 patients at a median follow-up of 11.4 years, with no differences found in terms of long-term weight loss between them. In the same study, both procedures reached an equal change in BMI of 17.8 and 18.1 kg/m^2^ in the group of superobese patients (>50 kg/m^2^) [15]. An early prospective randomized study by Brolin et al. with a shorter follow-up of 43 ± 17 months comparing Short-Limb Gastric Bypass (BPL: 15 cm; AL: 75 cm) to Long-Limb Gastric Bypass (BPL: 30 cm; AL: 150 cm) in 45 superobese patients found a significantly higher excess weight loss (EWL) in the long-limb group (64% vs. 50%) [16]. One may conclude that the longer BPL may also have affected patients’ weight loss in the long-limb group.

Another study suggesting that the length of the AL may be of less consequence to weight loss results than the length of the BPL or CL, was published by Risstad H. et al. They compared the standard RYGB (AL: 150 cm; BPL: 50 cm; CL: variable) to a Distal Gastric Bypass (AL: variable; BPL: 50 cm; CL: 150 cm) in a randomized controlled trial (RCT) with 113 patients and found no difference in weight loss after two years between the two groups [17]. The variable AL in the Distal Gastric Bypass group must have been very long indeed in these patients, which contradicts an effect of the AL on weight loss. 

Interestingly, another study by Süsstrunk J. et al. comparing a standard RYGB (AL: 150 cm; BPL: 60 cm; CL: variable) to a Very-Very Long-Limb Gastric Bypass (AL: variable; BPL: 60 cm; CL: 100 cm) in 232 patients after a follow-up of 9.4 years found a significant difference in weight loss and weight regain in favor of the Very-Very Long Limb Bypass. The authors reported no significant difference in the frequency of reoperations between both groups. However, while there were two patients with malnutrition/malabsorption in need of revisional surgery (reverse bypass) in the RYGB group, there were six patients suffering from malnutrition/steatorrhea that needed to be reversed (proximalization, reversed bypass) in the Very-Very Long-Limb Gastric Bypass group [18]. Considering the length of the CL in the Very-Very Long-Limb Gastric Bypass group (100 cm), fat malabsorption and possible loss of bile acids can be assumed in these patients. As Risstad et al.’s [17] and Süsstrunk et al.’s [18] studies appear to be quite similar in terms of surgical technique, the main reason for the difference in the outcomes seems to have been the length of the follow-up period. 

Another variation of RYGB relying on a long BPL is D-OAGB, which features the creation of a long and narrow pouch (similar to OAGB) and the exclusion of 150 cm of BPL. By performing a jejuno-jejunostomy, an AL of 70 cm is created to prevent backflow of biliary fluids to the pouch [19]. An RCT by Nergaard B. J. et al. compared a standard RYGB (AL: 150 cm; BPL: 60 cm; CL: variable) to a D-OAGB (AL: 60 cm; BPL: 200 cm; CL: variable) in 187 patients in terms of excess BMI loss. After seven years, a significant difference was achieved, with 78.4% of BMI loss in the long BPL group and 67.1% in the group with the long AL [20]. Darabi S. et al. studied the length of both the AL and BPL in RYGB after one and three years. Three hundred and thirteen patients in three groups (group 1: BPL 50 cm, AL: 150 cm; group 2: BPL: 150 cm, AL: 50 cm; group 3: BPL: 100 cm, AL: 100 cm) were compared. After one year no difference in %EWL was observed. However after three years patients with a longer BPL achieved a higher %EWL [21].

The length of the BPL also plays an important role in the postoperative development of deficiencies. Robert M. et al. [22] compared 129 OAGB (BPL: 200 cm) to 124 RYGB patients (BPL: 50 cm, AL: 150 cm) in an RCT with a noninferiority design. OAGB was not inferior to RYGB regarding weight loss and metabolic outcomes after two years. Nevertheless, 21.4% of severe nutritional complications in the OAGB group vs. none in the RYGB group (*p* = 0.0034) were found. Again, the main factor was the length of BPL, since no digestion takes place in this part, as opposed to the AL. Therefore, the risk of developing deficiencies after a standard RYGB is slightly lower than after OAGB [23].

Finally, a meta-analysis by Mahawar K. et al., which compared different limb lengths for RYGB, concluded that 100 cm to 200 cm of BPL + AL combined may lead to optimal results [24].

Thus, it may be concluded that the BPL length is more important in terms of weight loss and improvement of comorbidities than the length of the AL. Therefore, a D-OAGB may be superior to a standard RYGB. Nevertheless, if a BPL of more than 150 cm is considered, the total small bowel length should be measured intraoperatively to prevent deficiencies. 

## 4. One-Anastomosis Gastric Bypass

**Figure 2:**
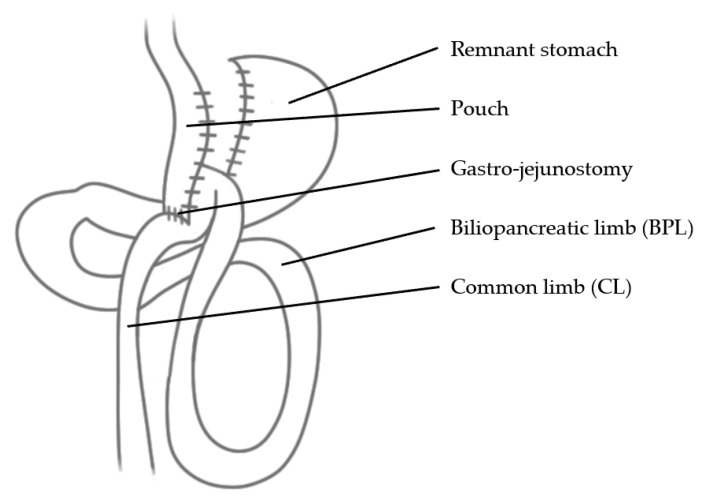
One-Anastomosis Gastric Bypass (OAGB).

OAGB (synonyms are Mini-Gastric Bypass or Omega-Loop Gastric Bypass) is an efficient and relatively safe bariatric procedure considering weight loss and postoperative development of deficiencies. As opposed to RYGB, OAGB does not feature an AL. Instead, it relies on a BPL and a CL [25] (Figure 2). It should be noted that (perhaps confusingly) a few publications refer to the CL as AL. 

OAGB was first described by Rutledge R. et al. in 2001 as a bariatric procedure in 1274 patients with a BPL length of 200 cm. The patients achieved an EWL of 77% after two years with a low complications rate [26]. Liagre A. et al. studied 115 patients eight years after OAGB with a 150 cm BPL and found an EWL of 84.8%. Interestingly, these results are not inferior to those mentioned above, despite the fact that a shorter BPL had been created. None of the patients were suffering from malnutrition, but high rates of Vitamin A and D deficiencies (54%; 33%) were found [27]. 

Several studies have compared different BPL lengths in OAGB patients to find an ideal balance between sufficient weight loss and a low risk of deficiencies. For example, comparing OAGB with a BPL of 150 cm to a BPL of 200 cm in 343 patients after two years, Boyle M. et al. found equal results in terms of EWL (74%; 75%). They reported no differences in albumin and hemoglobin levels [28]. In another study by Pizza F. et al., three groups of BPL lengths (150 cm, 180 cm, 200 cm) of 60 patients each were compared. After two years, no differences in terms of EWL, remission of type 2 diabetes (DMII) and arterial hypertension were found between the groups. Nevertheless, significant differences in iron and ferritin deficiencies were observed between the 150 cm and the 200 cm groups. Therefore, the authors concluded a BPL length of 150 cm–180 cm to be the safest and most effective option even in patients with a BMI > 50 g/m^2^) [29]. Ahuja A. et al. compared OAGB with BPL lengths of 150 cm, 180 cm and 250 cm. In the third group, 15% were reported to suffer from severe malnutrition and anemia [30]. Jedamzik J. et al. found that nutritional deficiencies were generally increased after OAGB with a tendency towards higher rates in longer BPL lengths, without improved weight loss [31].

By contrast, Charalampos M. et al. did not find any correlations between the BPL length (comparing 200 cm, 250 cm, and 300 cm BPL) and deficiencies after three years [32].

Some studies have compared a fixed BPL of 200 cm with a BPL of variable length. A recently published RCT by Nabil T. et al., for example, compared two groups of OAGB: group 1: BPL: 200 cm; group 2: CL: 400 cm (with a mean BPL of 301 cm). No significant differences in terms of weight loss were found, however, group 2 showed greater albumin, iron and hemoglobin deficiency rates [33]. Komaei I. et al. also compared fixed 200 cm BPL in the first group to a BPL of 40% of the small bowel in the second group and found less deficiencies of vitamins A, D, B12, iron and albumin one year after OAGB in the tailored BPL group, despite the fact that some patients in the tailored BPL group had a CL length of only 250 cm [34]. 

Lee W. J. et al. presented a different approach by tailoring BPL lengths to patients’ preoperative BMI. They compared these tailored BPL lengths (150 cm for BMI > 40 kg/m^2^, 250 cm for BMI 40–50 kg/m^2^, 350 cm for BMI > 50 kg/m^2^) in 644 patients. The mean BMI reduction was staggered as expected: 10.7, 15.5 and 23.5 kg/m^2^, respectively. Severe anemia was detected more frequently in the group with the lowest BMI [35]. 

In conclusion, the results of studies comparing different BPL lengths after OAGB are diverse. It appears that differences regarding the length of the BPL tend to impact the risk of deficiencies more strongly than weight loss and remission of comorbidities. Therefore, the recent IFSO Consensus Conference recommends a BPL of 150–180 cm for OAGB as effective and safe. If a BPL of more than 200 cm is created, the entire small bowel length should be measured intraoperatively to ensure that the CL is long enough [36]. A CL of at least 300 cm will likely prevent patients from developing malnutrition and deficiencies.

## 5. Single-Anastomosis Duodeno-Ileal Bypass with Sleeve Gastrectomy (SADI-S)

**Figure 3:**
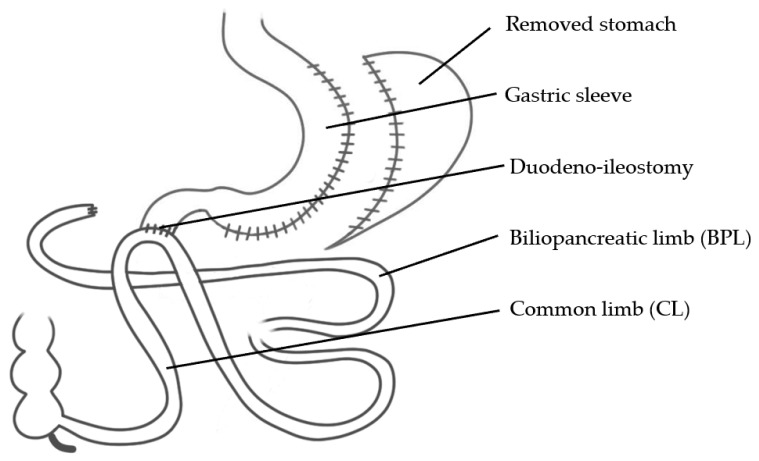
Single-Anastomosis Duodeno-Ileal Bypass + Sleeve Gastrectomy (SADI-S).

SADI-S (Figure 3) is a single-anastomosis procedure and may also be described as a combination of SG and a gastric bypass. Synonyms are: One-Anastomosis Duodenal Switch (OADS), Stomach Intestinal Pylorus Sparing (SIPS) or Loop-Duodenal Switch (Loop DS). After performing an SG, the duodenum is transected 3–4 cm post pylorus and an anastomosis is sutured between the duodenum and the ileum at a distance of 200–300 cm from the ileocecal valve [37]. The IFSO position statement of 2018 supported SADI-S as a recognized bariatric and metabolic procedure, even though studies with long-term follow-up in terms of safety and efficiency, as well as RCTs, have yet to be published [38]. 

By comparing the results of high-quality comparative studies on SADI-S, the aim of this chapter is to shed some light on the question: how much of the small bowel should remain in the food stream (CL) to maintain good weight loss results, yet keep the risk of deficiencies at an acceptable level? The development of the most effective and safe technique may, for example, be observed in a series of studies by A. Torres and A. Sanchez-Pernaute. Sanchez-Pernaute A. et al. first reported one to three years follow-up in 50 patients with a CL length of 200 cm. The EWL was 94.7% after one year and over 100% after three years, with 100% DMII remission and 91.3% remission of arterial hypertension. Patients with anemia (10%) and hypoalbuminemia (8%) after the first postoperative year recovered by the third year [37]. However, four patients (8%) had to be revised due to malnutrition, so that the authors then adjusted their technique by elongating the CL to 250 or 300 cm [39]. In a subsequent study with a total follow-up of up to four years, the next 50 patients were operated creating a 250 cm CL length and were analyzed together with the previously published patients. The EWL was stable >95% over the years with 90% DMII and 58% arterial hypertension remission rates [40]. 

In a more recent study of 65 patients after SADI-S (CL: 250 cm) and two years of follow-up, Moon R.C. et al. reported an EWL of 74.3% and 100% DMII remission rate. However, it was claimed that close monitoring of liver enzymes and nutritional status were necessary to avoid long-term complications [41]. The majority of studies published later performed a CL of 300 cm and also found an EWL between 70% and 90% with a low risk of malnutrition [42,43,44,45]. 

Midterm results of SADI-S are very satisfactory as well. Zaveri H. et al. published a large series of SADI-S with 300 cm CL in 437 patients at a four-year follow-up. The EWL after this period was 85.7% with 81.3% remission of DMII and 70.7% remission of arterial hypertension [46]. The most recent study on SADI-S was published by Surve A. et al. and included 750 SADI-S patients (CL: 300 cm) and a follow-up of six years. The authors reported 80.7% EWL and 77% DMII remission. Nutritional deficiencies were acceptable after five years. However, levels of albumin, total protein, calcium, parathormone and vitamin E had lowered significantly in these patients [47].

There are only a few case series reporting revisional procedures in cases of severe deficiencies or malnutrition after SADI-S. In those rare cases, revisions are lengthening of the CL or a conversion to RYGB. Horsley B. et al. collected nine cases of revisions (lengthening of the common limb) after SADI-S due to hypoproteinemia or chronic diarrhea. The CL before the revision was between 160 and 400 cm and was lengthened to 450–870 cm in the procedure. All patients with chronic diarrhea before the revision had normal bowel movement postoperatively, and the patients with hypoproteinemia improved their protein levels [48]. Vilallonga et al. published a case-series of five patients after SADI-S (CL between 170 and 250 cm). Some were converted to RYGB, others had lengthening of the CL due to malnutrition [49]. 

To conclude, SADI-S is one of the most effective bariatric procedures regarding weight loss and may be performed especially in patients with a very high BMI as well as patients with good compliance. One of the advantages of SADI-S is that the exact length of the small bowel in the food stream is known in each patient. The studies published so far have shown that a CL of 300 cm may achieve excellent %EWL with an acceptable risk of vitamin and micronutrient deficiencies [46]. Nevertheless, SADI-S is still a relatively new procedure and should therefore be performed in bariatric centers with good expertise and the capacity for sufficient aftercare.

## 6. Biliopancreatic Diversion (with Duodenal Switch)

BPD was first described in 1979 by Scopinaro N. et al. in 18 patients with different lengths of the CL [50]. In 1993 Marceau P. et al. published an alteration of this procedure named BPD-DS, which included the creation of a gastric Sleeve instead of a partial stomach resection. They compared 156 BPD-DS with a CL of 100 cm to patients with BPD and a CL of 50 cm, and found equal weight loss results but also a lower rate of undesirable side effects [51]. 

**Figure 4:**
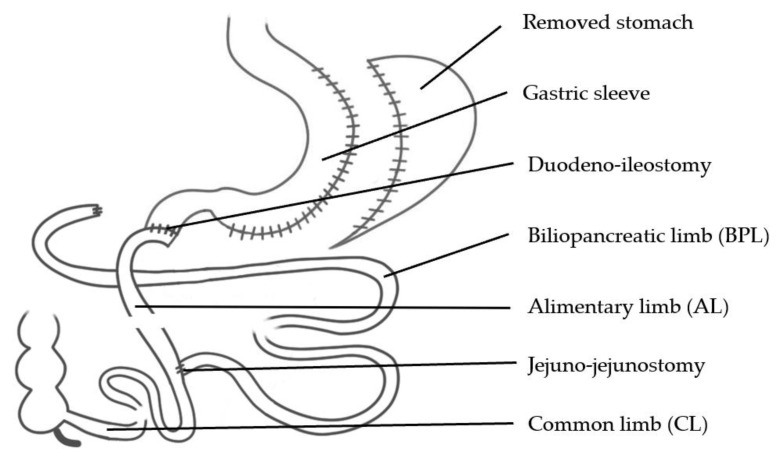
Biliopancreatic Diversion with Duodenal Switch (BPD-DS).

Studies on BPD-DS have commonly found very good to excellent weight loss results, yet high rates of deficiencies. However, a longer CL may lower the deficiency rates. An RCT by Lebel S. et al. comparing a classic BPD- DS (Figure 4) featuring 100 cm of CL to a variation with a CL of 200 cm initially, found similar weight loss results but significantly more weight regain in the 200 cm CL group. On the other hand, the second group had a lower albumin deficiency rate, lower hyperparathyroidism and a lower number of daily bowel movements [52]. 

Another RCT by Risstad H. et al., comparing a classic RYGB (BPL: 50 cm; AL: 150 cm) to a classic BPD-DS (AL: 200 cm; CL: 100 cm) in superobese patients >50 kg/m^2^ after five years, found greater weight loss and improvement of hyperlipidemia and glucose levels in BPD-DS, but also a higher rate of surgical, nutritional and gastrointestinal adverse events.

The results of long-term studies on BPD-DS are also quite homogenous. Again, they report excellent weight loss results but also high to extremely high rates of deficiencies. Topart P. et al. reported the outcome of BPD-DS after a follow-up of ten years. The EWL of 73% was excellent after this period. Nevertheless, high rates of fat-soluble deficiencies, hyperparathyroidism and a 14% revision rate due to nutritional complications were found [53]. Another long-term follow-up (>10 years) study by Bolckmans R. et al. also found excellent outcomes in terms of weight loss but at the cost of protein and nutritional deficiencies [54]. Finally, a long-term study nine years after BPD-DS reported dramatically high deficiency rates even in patients given adequate vitamin supplementation, as well as protein deficiencies in 30% of the patients and anemia in 40%. Therefore, the authors suggested continuous measurement of blood levels and clinical monitoring of these patients [55].

Despite the well-known potential of bariatric procedures to improve nonalcoholic steatohepatitis (NASH), there is a certain risk of total liver failure after strong malabsorptive procedures [56,57]. A nationwide Belgian survey collected ten patients listed for liver transplantation after bariatric surgery, nine of whom had BPD [58].

In conclusion, BPD is an operation with excellent results in terms of weight loss and remission of comorbidities, at the price of a high risk of postoperative deficiencies, malnutrition and adverse gastrointestinal events. As suggested by the studies referred to in this review, a CL length of only 100 cm may, in fact, be too short to equal a balanced operation. Therefore, BPD and BPD-DS currently only play a minor part as bariatric procedures worldwide. However, these procedures may be indicated for superobese patients. In any case, there is a clear contraindication of these methods for any noncompliant patient lacking lifelong commitment to vitamin supplementation.

## 7. Revisional Procedures for Weight Regain

Patients experience their nadir weight about 6–18 months after the bariatric procedure [22]. While most patients are able to maintain this weight, or experience a very slow reincrease in the long-term follow-up, about 5–10% suffer from significant WR, indicating a reoperation. On the one hand, a revisional procedure may include improving/re-establishing the restriction of the pouch (pouch-resizing, pouch-banding, restriction of the gastro-jejunostomy, etc.) or, on the other, malabsorption may be added [10].

Two studies described early experiences with WR operations. Sugerman H.J. et al. described five patients with a distalization for WR after RYGB and shortened the CL to only 50 cm. All patients developed malnutrition and were revised. Nevertheless, two of them died due to hepatic failure. In a second step, the CL was then increased to 150 cm in 22 patients. Besides good additional weight loss results, again, three patients were reoperated due to malnutrition. The authors concluded that a CL of only 50 cm meant an inacceptable level of morbidity and mortality, and that even patients with a CL of 150 cm would need continuous nutritional support thereafter. [59]. The other publication by Fobi M. et al. reported 65 patients that were converted from a Fobi pouch operation to a Distal Gastric Bypass for insufficient weight loss. Due to malnutrition in 15 patients, six of them had to be converted to a Short-Limb RYGB [60].

In a more recent study on WR after RYGB presented by Caruana J.A. et al., ten patients were bypassed more than 70% of the small bowel length and ten were bypassed less than 70%. The additional EWL was 47% in the first and 26% in the second group. However, the authors reported diarrhea in five patients and revision due to malnutrition in three patients of the first group [61]. Felsenreich et al. studied 30 patients who had shortening of the CL to only 100 cm due to WR after RYGB. Nine patients (30%) had to be reoperated (lengthening of the CL to 250 cm) for malabsorption in the follow-up [10].

Buchwald H. et al. published 53 patients that suffered from WR and insufficient weight loss after RYGB. In 47 patients, both the AL and CL were shortened to 75–100 cm, and in six patients the total length (CL + AL) was shortened to 250 cm. While the BMI decreased from 47.2 to 31.4 kg/m^2^ after five years, the complication rate was high, with 23 patients (43.4%) in need of total parenteral nutrition and 14 (26.4%) patients needing a revisional procedure [62]. 

In a current study, Ghiassi S. et al. presented 96 patients after three years. Patients with a total small bowel length of 400–450 cm were less likely to develop nutritional issues than patients with 250–300 cm in the food passage [63].

To conclude, adding malabsorption by decreasing the length of the CL should always be done very carefully. Patients chosen for this approach need to show very good compliance in terms of a commitment to vitamin supplementation and routine aftercare. It is important that these patients do not suffer from dysphagia or vomiting due to a stenosis or ulcer of the gastro-jejunostomy, as these increase the risk of postoperative malnutrition [57]. In every reoperation for weight regain, the entire small bowel must be measured and documented [64]. The CL, or the CL + AL lengths, respectively, should not be shortened below 300–350 cm to minimize the risk of deficiencies and malnutrition [65,66]. 

## 8. Conclusions

It is hard to assess how much malabsorption the individual patient needs; as we have seen, the length of the small bowel differs in each individual. It is necessary to find a balance between sufficient weight loss and remission of comorbidities on the one hand, and a low risk of possible deficiencies and malnutrition on the other. The larger the part of the small bowel removed from the food stream, the higher the importance of the patient’s compliance with daily vitamin supplementation as well a thorough aftercare program. 

In fact, the reasoning behind the choice of procedure varies to a great extent worldwide. However, one may use the following suggestions as a possible algorithm for choosing an appropriate malabsorptive procedure: Patients suffering from gastro-esophageal reflux disease may benefit most from RYGB, whereas SADI-S or BPD-DS are recommended for super-obese patients (>50 kg/m^2^) and OAGB may be performed in any patients without reflux. 

Studies have pointed out that the effect of a BPL (in RYGB or BPD-DS) removed from the food stream completely has more impact than an AL, where protein and carbohydrate digestion is continued. In patients with a BPL longer than 150 cm, the entire small bowel should be measured to ensure that the CL is long enough. A rule of thumb for any malabsorptive procedure should be maintaining at least 300 cm of small bowel (CL or CL + AL) in the food stream to prevent the development of deficiencies and malnutrition.

If nutritional deficiencies and malabsorption cannot be treated conservatively in an adequate way, a reoperation should be considered early on to increase the length of small bowel in the CL. In patients with a critical body condition, the placement of a flow-care tube to the remnant stomach (in OAGB or RYGB) may be considered as a first step to start enteral nutrition of the BPL.

The initial question, “how much malabsorption do we need?”, cannot be answered in definitive terms but must be answered for each patient individually, as it is multilayered and depends on several individual factors, such as preoperative weight, comorbidities and the patient’s compliance.
